# The Conjunctival Turnover: A Simple and Effective Method for Sclero-Corneal Protection in Pre-Septal Transconjunctival Approach to the Orbital Floor

**DOI:** 10.7759/cureus.18884

**Published:** 2021-10-19

**Authors:** Anantanarayanan Parameswaran, Elavenil Panneerselvam, Naveenkumar Jayakumar

**Affiliations:** 1 Oral and Maxillofacial Surgery, Meenakshi Ammal Dental College, Chennai, IND; 2 Oral and Maxillofacial Surgery, SRM Dental College and Hospital, Ramapuram Campus, Chennai, IND; 3 Oral and Maxillofacial Surgery, Sri Ramachandra Medical College and Research Institute, Chennai, IND

**Keywords:** complications in orbital surgery, corneal abrasion, sclera-corneal protection, corneal shield, corneal protection

## Abstract

Transconjunctival approaches have become the mainstay for most surgeons performing orbital wall reconstructions. Adequate care needs to be exercised for the protection of the cornea and sclera during these surgeries as they may involve placement of grafts or implants in situ apart from the routine intra-orbital dissections. The authors describe a simple technique of developing a conjunctival turnover flap for sclero-corneal protection in transconjunctival approaches to the orbit.

## Introduction

The transconjunctival approach is one of the most preferred incisions to expose the orbital floor and the infra-orbital rims. The use of corneal protectors/shields is a must for protecting the cornea from iatrogenic injuries during intra-orbital surgery. Traditionally, shields are made of plastics, silicone, or metals. The authors describe a simple and effective method of using the palpebral conjunctiva as a turnover flap for sclero-corneal protection in transconjunctival approach.

Corneal abrasions and epithelial defects are complications associated with peri-orbital surgery when optimal corneal protection is not ensured with a reported incidence of 0.01-0.11% [1]. Though self-limiting, this is often a painful and irritating sequel to most afflicted patients [1]. Common methods of preventing such inadvertent injuries include use of temporary tarsorrhaphy, “frost sutures,” corneal shields, and ophthalmic visco-elastic devices (OVD) [2]. However, the inherent disadvantages of these methods include restriction of direct visualization and easy access to the globe and/or hindrance to intra-operative monitoring of pupillary reflexes [2]. Devices like the OVDs may also not be economically viable for routine use [3].

The authors advocate utilizing a conjunctival flap elevated from the palpebral conjunctiva during a pre-septal transconjunctival approach, as a temporary corneal protector during the exposure and dissection of the orbital floor for reconstructive surgery.

## Technical report

**Figure 1:**
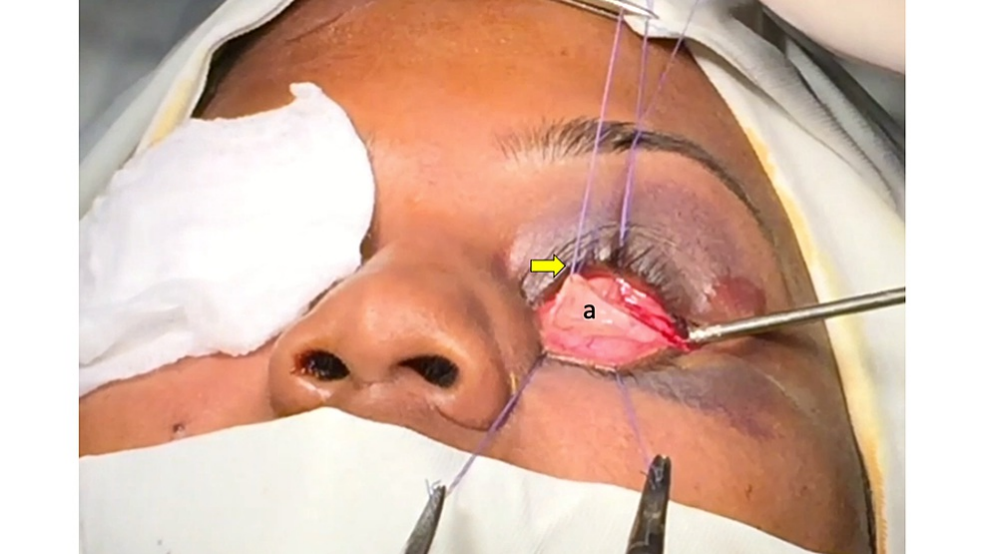
Intra-operative picture demonstrating elevated palpebral conjunctival turnover flap (a) and placement of retraction sutures (yellow arrow).

**Figure 2:**
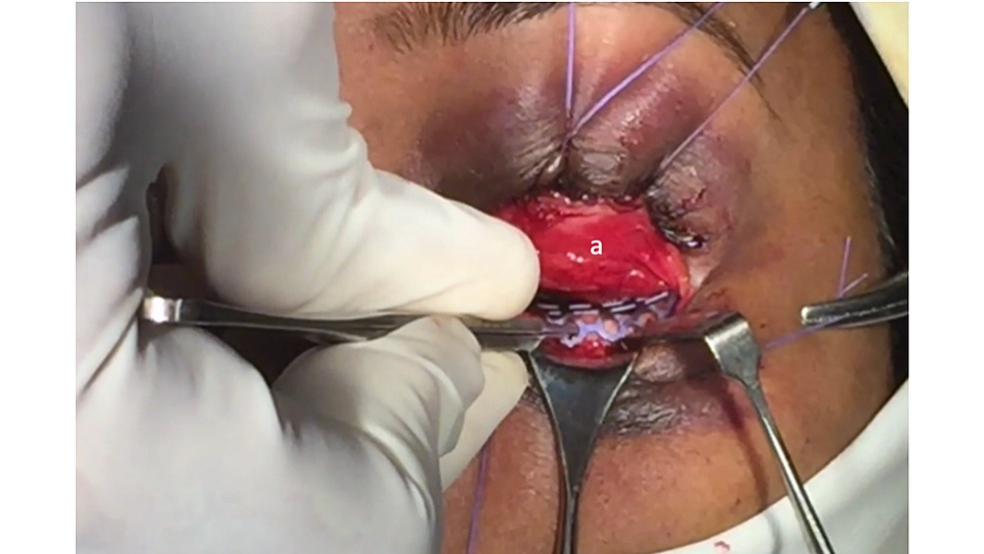
Picture of the turnover flap (a) providing excellent protection to the cornea and sclera during implant placement and fixation.

Infiltration of 2cc of lignocaine hydrochloride and adrenaline (1 in 100,000) is performed for achieving hemostasis prior to incision. Sutures with 4-0 silk are placed on either side of the lid margin to provide retraction for the incision. The authors prefer to use lateral cantholysis with the transconjunctival incision if the orbital defect is large. The incision on the palpebral conjunctiva is made using microneedle electrocautery at an infra-tarsal level. A flap is then raised in a sub-conjunctival fashion extending to the bony margin in a pre-septal plane. This creates a conjunctival flap that is large, elastic, and reasonably resistant to tears. Traction sutures are placed on the conjunctival flap and draped over the exposed part of the globe, held on by small hemostats, or may be clipped to the head drape. This provides excellent cover to the corneal/scleral tissue, protecting them from any injuries that may occur intra-operatively due to instrumentation. A coating of lubricants such as hydroxypropyl methylcellulose (HPMC) gel may also be applied over the cornea and globe prior to draping of the conjunctival flap which offers additional protection while preventing dryness of the cornea (Figures 1, 2).

## Discussion

Intra-orbital surgical procedures are associated with the risk of corneal injury. The etiology of such damage may be physical, mechanical, or chemical. Physical causes include changes in temperature and air within the operation theatre which inflict indirect trauma to cornea [4]. Mechanical reasons may be inadvertent direct trauma due to instruments or undue pressure exerted on the globe [5]. Chemical injury is commonly due to anti-microbial agents [6].

Protection of the eye during non-orbital surgeries is different from protection of eye during orbital surgery. The safety measures and options for non-orbital surgeries are numerous as indicated by literature, such as passive closure of eyelids with adhesive tapes, eye ointments, tarsorrhaphy, protective goggles, hydrogel, or bio-occlusive dressings [7,8]. But there is sparse literature regarding the protection of eye during orbital surgeries. Ointments, scleral shell, or corneal shields are the cited preferences [9]. However, they are associated with numerous limitations. Ointments by virtue of their thin layer do not provide an effective barrier to physical insults. Scleral shell or shields are firm and hence preclude free movement of the eyelid or tissues around the globe during surgery. Further, scleral shells do not allow intra-operative monitoring of the status of pupil.

The “conjunctival turnover” flap proposed by the author uses a single incision to expose the surgical site (floor of the orbit) as well as provide an effective cover to protect the globe in the intra-operative phase. The authors have been using this technique for more than 10 years with excellent results and intra-operative convenience at no additional costs. The advantages of this technique over the conventional methods include the fact that the flap can be turned down anytime the surgeons need to evaluate pupillary reflexes or the globe for comparing projection (enophthalmos correction) with the contralateral side during primary reconstructive or revision surgery. It also negates the negatives of shields/conformers which may produce pressure/abrasions when handled injudiciously or with repeated removal and reapplications. Unlike the synthetic shields which may elicit allergic reactions, the conjunctival flap is derived from one’s own native tissue. This flap also has an additional advantage of ensuring adequate protection of the cornea as well as the sclera because of the wide area of coverage. The flap conforms to the shape and position of the globe in a smooth manner. However, this technique cannot be used in post septal approach to the orbit.

The clinical effectiveness of the technique may be further validated by conducting a randomized controlled trial of the author's technique in comparison with the scleral shell.

## Conclusions

The conjunctival turnover is a simple, effective, and reproducible method to achieve sclero-corneal protection during orbital surgery, with the advantage of not requiring any additional armamentarium or pharmacological agents. The technique involves no added costs and easily accommodates for effective evaluation of ocular structures and reflexes intra-operatively. This is a significant advantage in orbital surgery which requires periodic peri-operative evaluation of ocular function.

## References

[1] Malafa MM, Coleman JE, Bowman RW, Rohrich RJ (2016). Perioperative corneal abrasion: updated guidelines for prevention and management. Plast Reconstr Surg.

[2] Kittur MA, Isaac R, Parkin IR (2013). Physiological method of corneal protection during periocular surgery. Br J Oral Maxillofac Surg.

[3] Naylor SG, Dunleavy D, Kolb S (2013). A cheaper method of corneal protection during periocular surgery: in response to: Kittur MA, Isaac R, Parkin IR. Physiological method of corneal protection during periocular surgery. Br J Oral Maxillofac Surg 2013;51:178-9. Br J Oral Maxillofac Surg.

[4] Snow JC, Kripke BJ, Norton ML, Chandra P, Woodcome HA (1975). Corneal injuries during general anesthesia. Anesth Analg.

[5] Keita H, Devys JM, Ripart J (2017). Eye protection in anaesthesia and intensive care. Anaesth Crit Care Pain Med.

[6] Roth S, Thisted RA, Erickson JP, Black S, Schreider BD (1996). Eye injuries after nonocular surgery. A study of 60,965 anesthetics from 1988 to 1992. Anesthesiology.

[7] Grover VK, Kumar KV, Sharma S, Sethi N, Grewal SP (1998). Comparison of methods of eye protection under general anaesthesia. Can J Anaesth.

[8] Anderson DA, Braun TW, Herlich A (1995). Eye injury during general anesthesia for oral and maxillofacial surgery: etiology and prevention. J Oral Maxillofac Surg.

[9] Ogle CA, Shim EK, Godwin JA (2009). Use of eye shields and eye lubricants among oculoplastic and Mohs surgeons: a survey. J Drugs Dermatol.

